# CSDE1 Associates with TOM20 and Mitochondrial Protein-Encoding mRNAs in Sensory Neurons

**DOI:** 10.3390/antiox15050608

**Published:** 2026-05-11

**Authors:** Hoyong Jin, Eunsu Jang, Eunhye Park, Ju Yeon Lee, Ju Hwan Song, Yongcheol Cho

**Affiliations:** 1 Department of Brain Sciences, Daegu Gyeongbuk Institute of Science and Technology (DGIST), Daegu 42988, Republic of Korea; jhn1462@dgist.ac.kr (H.J.); tmvprxj33@dgist.ac.kr (E.J.); eun3705@dgist.ac.kr (E.P.); 2 Biopharmaceutical Mass Spectrometry Research Unit, Korea Basic Science Institute (KBSI), Ochang 28119, Republic of Korea; jylee@kbsi.re.kr (J.Y.L.); sjhs5904@kbsi.re.kr (J.H.S.); 3 Department of Bio-Analytical Science, University of Science and Technology (UST), Daejeon 34113, Republic of Korea; 4 Department of Biotechnology, Graduate School, Korea University, Seoul 02841, Republic of Korea

**Keywords:** CSDE1, TOM20, mitochondrial proteostasis, RNA-binding proteins, mitochondrial mRNA, protein import, OXPHOS, sensory neurons, translation-import coupling, oxidative stress

## Abstract

Mitochondrial proteostasis in neurons relies on the coordinated expression, targeting, and import of a predominantly nuclear-encoded proteome to meet high metabolic demands. Here, we identify the RNA-binding protein cold shock domain containing E1 (CSDE1) as a TOM20-associated factor linked to mitochondrial protein-encoding mRNAs in sensory neurons. CSDE1 immunoprecipitation followed by sequencing from naïve dorsal root ganglion tissue revealed association with nuclear-encoded mitochondrial mRNAs enriched for inner membrane/matrix and oxidative phosphorylation pathways. A subset of CSDE1 localized to mitochondria and associated with the outer mitochondrial membrane import receptor TOM20 via its N-terminal region in an RNA-independent manner. In cultured sensory neurons, CSDE1 depletion reduced the mitochondrial-fraction abundance of representative nuclear-encoded electron transport chain mRNAs and decreased the abundance of selected mitochondrial proteins in the mitochondrial fraction. CSDE1 depletion reduced TMRM-positive mitochondrial puncta density along sensory neurites, without significantly increasing MitoSOX-detectable mitochondrial superoxide signals under either basal or oxidative challenge conditions. These findings identify CSDE1 as a TOM20-associated RNA-binding protein linked to mitochondrial protein-encoding transcripts in sensory neurons and support a model in which CSDE1 contributes to mitochondria-associated post-transcriptional regulation.

## 1. Introduction

Neurons face exceptional bioenergetic and proteostatic challenges because their extended morphology imposes long-distance constraints on the supply and maintenance of essential organellar components. Mitochondria are central to neuronal physiology, supporting ATP production, calcium buffering, and signaling, and they must be maintained across spatially dispersed subcellular compartments such as distal axons, presynaptic terminals, and growth cones. Accordingly, neuronal mitochondrial function relies on the coordinated expression, targeting, import, and assembly of a proteome that is overwhelmingly encoded in the nuclear genome and synthesized by cytosolic ribosomes.

The biogenesis of nuclear-encoded mitochondrial proteins is coordinated with the mitochondrial import machinery [1,2,3,4,5,6]. Following translation in the cytosol, precursor proteins must remain in an import-competent state, be recognized at the mitochondrial surface, be translocated through the translocase of the outer mitochondrial membrane (TOM) complex, and subsequently be sorted and assembled by downstream machineries. Among TOM components, the import receptor TOM20 plays a pivotal role in early substrate recognition by binding N-terminal targeting presequences and facilitating delivery of precursors to the translocation pore [7,8,9,10,11]. In parallel with classical post-translational import models, accumulating evidence supports a spatially organized translation-translocation framework in which subsets of nuclear-encoded mitochondrial mRNAs and/or ribosomes are positioned near the outer mitochondrial membrane, potentially enabling localized translation and efficient transfer to import receptors [1,9,12,13,14,15,16,17]. This organization may be particularly relevant in neurons, where localized protein synthesis could help sustain compartmentalized mitochondrial maintenance when long-range transport from the soma is limiting [18,19].

Despite these advances, the molecular mechanisms that coordinate mitochondrial mRNA selection, subcellular mRNA positioning, and engagement of the import apparatus remain incompletely understood. RNA-binding proteins (RBPs) could provide an important regulatory interface. By binding mRNA cohorts and simultaneously associating with outer-membrane import components, RBPs could promote transcript enrichment near the mitochondrial surface and contribute to localized ribonucleoprotein (RNP) organization. However, relatively few RBPs have been linked to the TOM complex that connects selective mRNA association with the import gate.

CSDE1 (cold shock domain-containing protein E1; also known as UNR) is a multifunctional RBP implicated in diverse post-transcriptional processes, including the organization of transcript cohorts and regulation of translation in context-dependent manners [20,21,22,23]. Here, we examined whether CSDE1 may participate in a mitochondria-associated RNA regulatory pathway in sensory neurons. Using TOM20 as bait in proteomic interaction screens, we identified CSDE1 as a TOM20-associated RBP. By CSDE1 immunoprecipitation followed by sequencing from naïve dorsal root ganglion (DRG) tissue, we found that CSDE1-associated transcripts were enriched for nuclear-encoded mitochondrial mRNAs linked to inner membrane/matrix and oxidative phosphorylation pathways. We further found that a distinct fraction of CSDE1 localizes to mitochondria and associates with TOM20 via its N-terminal region in an RNA-independent manner. Finally, by combining mitochondrial fractionation with transcriptome analysis in cultured sensory neurons, we observed that CSDE1 depletion reduced the mitochondrial-fraction abundance of nuclear-encoded mitochondrial mRNAs, including respiratory-chain-related transcripts. Together, these findings support a model in which CSDE1 is associated with selective mitochondrial mRNA regulation and the TOM20-dependent import interface in sensory neurons.

## 2. Materials and Methods

### 2.1. Embryonic DRG Neuron Cultures

All procedures involving mice were conducted in accordance with the guidelines approved by the Institutional Animal Care and Use Committee (IACUC) at Daegu Gyeongbuk Institute of Science and Technology (DGIST-IACUC-25042502-0005). Mice were housed in a pathogen-free facility under a 12 h light/dark cycle with ad libitum access to food and water. Surgical procedures were performed under anesthesia using 3% isoflurane in oxygen. All surgical instruments and the incision site were sterilized prior to the procedure. For embryonic DRG (eDRG) cultures, DRGs were isolated from E13.5 CD-1 mouse and dissociated in 0.05% trypsin-EDTA. The dissociated cells were plated onto culture dishes coated with poly-D-lysine and laminin and maintained in Neurobasal Medium (Gibco, Grand Island, NY, USA), supplemented with 2% B-27 (Gibco), 1% GlutaMAX, 1 μM 5-fluoro-2′-deoxyuridine (Sigma, St. Louis, MO, USA), 1 μM uridine (Sigma), 1% penicillin-streptomycin, and 50 ng/mL 2.5S nerve growth factor (Envigo, Indianapolis, IN, USA, BT-5017). Cultures were incubated at 37 °C in a humidified atmosphere with 5% CO_2_ [24,25].

### 2.2. Lentiviral Constructs

Mouse CSDE1 was knocked down in embryonic dorsal root ganglion (DRG) neurons using lentivirus-mediated gene delivery. A target sequence specific to the 3′ untranslated region (3′UTR) of mouse Csde1 (TRCN0000276864; target sequence: GCGCCTTATAGTGTCAATTTA) was subcloned into the pLKO vector. Lentiviruses were produced using the Lenti-X Packaging Single Shots (Takara, Kusatsu, Japan, 631276) system. For in vitro transduction, the lentivirus was applied to embryonic DRG neurons at DIV2 in culture medium [26].

### 2.3. Antibodies and Western Blot Analysis

The following antibodies were used in this study: anti-glyceraldehyde 3-phosphate dehydrogenase (GAPDH) antibody (Santa Cruz Biotechnology, Dallas, TX, USA, sc-32233, clone 6C5, 0.2 mg/mL, 1:200), anti-alpha tubulin antibody (Santa Cruz Biotechnology, sc-53030, 0.2 mg/mL, 1:200), anti-Cold shock domain-containing protein E1 (CSDE1) antibody (Abcam, Cambridge, UK, ab201688, 1:1000), anti-HA tag antibody (Santa Cruz Biotechnology, sc-7392), anti-Translocase of the Outer Mitochondrial Membrane 20 (TOM20) antibody (Cell Signaling Technology, Danvers, MA, USA, #72610, 1:1000), and the Electron Transport Chain (Complex I, III, IV) Antibody Sampler Kit (Thermo Fisher Scientific, Waltham, MA, USA, #42642).

For biochemical analysis, cultured eDRG neurons were harvested at DIV6 and lysed in 1× SDS lysis buffer containing 63 mM Tris (pH 6.8), 2% SDS, and 10% glycerol. Protein concentrations were measured using the DC Protein Assay (Bio-Rad, Hercules, CA, USA, 5000116) with bovine serum albumin (BSA) as the standard. Equal amounts of protein were separated by SDS-PAGE and transferred to a nitrocellulose membrane. Membranes were blocked with 5% non-fat dry milk in Tris-Buffered Saline (TBS) containing 0.1% Tween-20 (TBS-T) for 1 h, followed by overnight incubation at 4 °C with primary antibodies. After washing five times with TBS-T, membranes were incubated with secondary antibodies for 1 h and again washed five times with TBS-T. Protein expression levels were analyzed using enhanced chemiluminescence (ECL) and detected with the iBright imaging system (Thermo Fisher Scientific) [27].

### 2.4. Co-Immunoprecipitation and Liquid Chromatography-Tandem Mass Spectrometry (LC-MS/MS)

To investigate protein–protein interactions, HEK293T cells were transfected with HA-tagged CSDE1 using polyethylenimine (PEI; Merck, Darmstadt, Germany, 408727) [28]. Cells were harvested and lysed in an immunoprecipitation (IP) buffer containing 20 mM Tris-HCl (pH 7.5), 150 mM NaCl, 1% NP-40, 1 mM EDTA, supplemented with a protease and phosphatase inhibitor cocktail (Roche). HA-tagged CSDE1 was immunoprecipitated from the lysates using Pierce™ Anti-HA Magnetic Beads (Thermo Fisher Scientific, 88836) at 4 °C for 16 h. The immunoprecipitates were then washed five times with IP buffer containing protease inhibitors, and the proteins were analyzed by SDS-PAGE followed by Western blotting [29].

To assess whether the interaction between TOM20 and CSDE1 is RNA-dependent, the immunoprecipitated complexes were incubated with RNase A (Roche, Basel, Switzerland, 10109142001, 0.1 mg/mL) and RNase T1 (Thermo Fisher Scientific, EN0541, 4 U/μL) at room temperature for 20 min. To identify the domain of CSDE1 responsible for the interaction with TOM20, HA-tagged partial CSDE1 constructs, each encoding different domains, were similarly subjected to immunoprecipitation.

To identify proteins interacting with TOM20, HEK293T cells were transfected with FLAG-tagged TOM20 using PEI, and immunoprecipitation was carried out as described above. The immunoprecipitated samples were separated by SDS-PAGE and visualized by SimplyBlue™ SafeStain (Thermo Fisher Scientific, LC6060). Protein bands were excised from the gel and subjected to LC-MS-based proteomic analysis at the Korea Basic Science Institute (KBSI) [30].

### 2.5. Proteomic Analysis

The reagents and resources used for the proteomic analysis are listed in Table 1.

**Table 1:** Reagents and resources used for the proteomic analysis..

Reagent or Resource	Source	Identifier
Chemicals		
Iodoacetamide (IAA)	Sigma-Aldrich	Cat # I1149
1,4-dithiothreitol (DTT)	Sigma-Aldrich	Cat # 43819
Formic acid (FA)	Merck	Cat # 5.33002
Ammonium bicarbonate (ABC)	Sigma-Aldrich	Cat # A6141
Trypsin	Promega	Cat # V5280
Methanol (MeOH; HPLC grade)	J.T Baker	Cat # 1.00030.4000
Acetonitrile (ACN; HPLC grade)	Merck	Cat # 1.00030.4000
Deionized water (DW)	Merck	Cat # 1.15333.2500

### 2.6. In-Gel Digestion for Mass Spectrometry

Protein samples were separated by SDS-PAGE on 10% acrylamide gels. The target protein bands were sliced from the gel, de-stained with 30% MeOH (*v*/*v*) and 50% ACN (*v*/*v*) in 10 mM ammonium bicarbonate (ABC) buffer, and dried. For reduction, sliced bands were incubated in 10 mM dithiothreitol (DTT) in 100 mM ABC buffer at 55 °C for 1 h. Alkylation was performed by incubation with 55 mM iodoacetamide (IAA) in 100 mM ABC buffer in the dark at room temperature for 40 min. Each band was washed sequentially with deionized water and 100% ACN and then dried. Proteins from each band were digested by trypsin in 50 mM ABC buffer and incubated at 37 °C for 16 h. The digestion was quenched by adding 50% ACN (*v*/*v*) containing 0.1% FA (*v*/*v*). Finally, the peptides were recovered by extraction once with 100% ACN and twice with 50% ACN (*v*/*v*) containing 0.1% FA (*v*/*v*). All extracted peptides were pooled for each sample and dried. The dried peptides were re-dissolved in 0.1% FA (*v*/*v*) in water prior to LC-MS/MS analysis.

### 2.7. Liquid Chromatography Tandem Mass Spectrometry

Extracted peptides were analyzed using a LC-MS/MS system consisting of an UltiMate 3000 RSLnano System (Thermo Fisher Scientific) and an Orbitrap Fusion Lumos mass spectrometer (Thermo Fisher Scientific) with a nano-electrospray source (EASY-Spray Sources) at KBSI. Peptides were first trapped in a trap column (2 cm × 75 μm, 3 μm, PepMapTM 100 C18 LC Column, Thermo Fisher Scientific, #164535) and then separated on an EASY-SprayTM HPLC Column (500 mm × 75 μm, 2 μm, Thermo Fisher Scientific, #ES903) at a flow rate of 250 nL/min. The mobile phases were composed of 100% water (A) and 100% acetonitrile (B), each containing 0.1% formic acid. The LC gradient began with 5% B, ramped to 5% B over 6 min, increased to 7% for 9 min, followed by 25% B over 75 min, and increased to 30% B for 10 min and to 95% B over 1 min, then was held constant for 8 min, and ended with 5% B over 1 min. After a gradient, the column was re-equilibrated with 5% B for 10 min before the next run. The voltage applied to produce an electrospray was 1800 V. The Orbitrap Fusion Lumos was operated in data-dependent mode, automatically switching between MS and MS/MS with a 3 s cycle time. Full scan MS spectra (400–2000 *m*/*z*) were acquired with a maximal ion injection time of 50 ms at a resolution of 120,000 and an automatic gain control (AGC) target value of 4.0 × 10^5^. MS/MS spectra were acquired at a resolution of 30,000 with a high-energy collision dissociation (HCD) of 27% normalized collision energy within a 1.2 Da isolation window. The AGC target value was 5.0 × 10^4^ with a maximal ion injection time of 54 ms. The exclusion time for previously fragmented ions was 30 s within 10 ppm.

### 2.8. Protein Identification

The Integrated Proteomics Pipeline using built-in search engines (IP2; Integrated Proteomics Applications, version 6.5.5, Inc., San Diego, CA, USA) was utilized for data analysis with the UniProt human protein database (released on 5 June 2025). The reversed sequences of all proteins were appended into the database for calculation of false discovery rate (FDR). ProLucid was used to identify the peptides, with a precursor mass error of 5 ppm and a fragment ion mass error of 50 ppm [31]. Trypsin was selected as the enzyme, with one potential missed cleavage. Carbamidomethylation at cysteine was chosen as a static modification. Oxidation at methionine was chosen as a variable modification.

### 2.9. Mitochondria Isolation

Mitochondria were isolated according to the manufacturer’s instructions using mitochondrial isolation reagents (Thermo Fisher Scientific, 89874 and 89801). Briefly, harvested HEK293T cells (2 × 10^6^ cells) or cultured eDRG neurons (1 × 10^6^ cells) were washed with PBS and lysed using mitochondria isolation reagent for cultured cells supplemented with protease inhibitor cocktail (Roche). The lysates were centrifuged at 700× *g* for 10 min at 4 °C to remove cell debris and nuclei. The resulting supernatant was transferred to a new tube and further centrifuged at 12,000× *g* for 15 min at 4 °C to separate the mitochondrial pellet from the cytosolic supernatant. The cytosolic fraction (supernatant) was subjected to trichloroacetic acid (TCA) precipitation to obtain cytosolic proteins. The mitochondrial pellet was lysed in 1× SDS lysis buffer containing 63 mM Tris (pH 6.8), 2% SDS, and 10% glycerol. For embryonic brain samples, brains were isolated from E13.5 mouse embryos, and four embryonic brains (~20 mg total) were used per experiment. The tissues were washed with PBS, cut into small pieces, and homogenized in PBS using a Dounce homogenizer. The homogenates were then lysed using a mitochondria isolation reagent for tissues supplemented with a protease inhibitor cocktail (Roche). Subsequent fractionation steps were performed as described above for cultured cells, yielding cytosolic and mitochondrial protein fractions [32,33].

### 2.10. RNA Sequencing and Analysis

To investigate the RNA pool bound to CSDE1 in dorsal root ganglion (DRG) neurons, lumbar 4 and 5 (L4 and L5) DRG tissues were harvested from mice for RNA extraction. To immunoprecipitate CSDE1 from DRG tissue, the tissues were homogenized in a lysis buffer containing 20 mM Tris-HCl (pH 7.5), 150 mM NaCl, 0.1% Triton X-100, 1 mM EDTA, RiboLock RNase Inhibitor (Thermo Fisher Scientific, EO0382), and supplemented with a protease and phosphatase inhibitor cocktail (Roche). Endogenous CSDE1 was immunoprecipitated using an anti-CSDE1 antibody prebound to Dynabeads Protein A (Thermo Fisher Scientific, 10002D). RNA was then extracted from the immunoprecipitates using the RNeasy Mini Kit (QIAGEN, Hilden, Germany, 74104).

To examine whether CSDE1 regulates mitochondria-associated transcripts in sensory neurons, RNA sequencing was also performed on mitochondrial RNA isolated from cultured embryonic DRG (eDRG) neurons. CSDE1 was knocked down in cultured eDRG neurons using lentivirus-mediated gene delivery as described above, while control neurons were infected with lentivirus containing the empty pLKO vector. After culture and infection, mitochondria were isolated from eDRG neurons using a reagent-based isolation method as described in Section 2.9. The mitochondrial pellet was collected, and total RNA was extracted using the RNeasy Mini Kit (QIAGEN, 74104). Independent biological replicates were generated by repeating this procedure three times.

Libraries were prepared using the Nanopore PCR-cDNA Barcoding Kit (Oxford Nanopore Technologies, Oxford, UK, SQK-PCS111), and sequencing was performed on a MinION device (Oxford Nanopore Technologies, software version 20.10.3). Real-time basecalling was conducted using the integrated basecaller Guppy [34]. Sequencing reads were mapped to the reference Mus musculus genome (GRCm38.p6, GCA_000001635.8) using Minimap2 (2.26-r1175) [35,36]. For complementary short-read RNA-seq analysis, libraries were sequenced on the DNBSEQ-G400 platform. Quality control-passed reads were quantified using Salmon against an index built from the same reference transcriptome, and transcript-level abundances were summarized to gene-level counts using tximport (1.40.0) for downstream differential expression analyses [37,38].

### 2.11. Gene Set Enrichment Analysis (GSEA)

Gene Set Enrichment Analysis (GSEA) was performed using our previously generated CSDE1 RIP-seq dataset from naïve adult mouse L4/5 dorsal root ganglion (DRG) tissue (GSE301321). For gene set ranking, gene-level abundance values from the CSDE1 immunoprecipitated RNA pool were used, and genes were ranked according to mean logCPM values after removal of missing values. The ranked gene list was generated using Ensembl gene identifiers and sorted in decreasing order. GSEA was performed in R (4.6.0) using the clusterProfiler package (4.20.0) with the gseGO function [39] against Gene Ontology (GO) categories for Cellular Component (CC) and Biological Process (BP) [40], using org.Mm.eg.db as the annotation database and ENSEMBL as the key type. The following parameters were applied: minGSSize = 5, maxGSSize = 500, pvalueCutoff = 0.05, and Benjamini–Hochberg (BH) multiple testing correction. To reduce redundancy among enriched GO terms, semantic similarity-based clustering was performed using the rrvgo package (1.24.0) [41]. Pairwise GO term similarity was calculated with calculateSimMatrix using the “Rel” method, and redundant GO terms were reduced using reduceSimMatrix with a threshold of 0.95 [42,43]. The final GSEA result set after redundancy reduction was used for downstream visualization and interpretation. For visualization, GeneCount was defined as the number of genes in the core_enrichment field for each GO term, and GeneRatio was calculated as GeneCount/setSize. Dot plots were generated from the reduced GSEA results, and the top categories were selected based on normalized enrichment score (NES). In the main figures, BP and CC categories relevant to mitochondrial pathways are shown.

### 2.12. Proximity Ligation Assay

Proximity ligation assay (PLA) was performed according to the manufacturer’s Duolink In Situ Fluorescence protocol (Sigma-Aldrich) [29]. Cultured embryonic DRG (eDRG) neurons were washed with phosphate-buffered saline (PBS) and fixed with 4% paraformaldehyde for 15 min at room temperature. Samples were permeabilized with PBS containing 0.1% Tween-20 (PBS-T) for 10 min and blocked with Duolink blocking solution at 37 °C for 1 h. Anti-CSDE1 (Abcam, ab201688, 1:300) and anti-TOM20 (Abcam, ab56783, 1:500) primary antibodies were applied overnight at 4 °C. Samples were then incubated with Duolink PLA probes anti-rabbit PLUS (Sigma, DUO92002) and anti-mouse MINUS (Sigma, DUO92004) for 1 h at 37 °C. Ligation was performed using Duolink ligation solution for 30 min at 37 °C, followed by rolling-circle amplification with Duolink amplification solution for 100 min at 37 °C according to the manufacturer’s instructions. After amplification, samples were subjected to immunostaining. Samples were blocked with PBS-T containing 5% goat serum and incubated overnight at 4 °C with anti-βIII-tubulin (TUJ1; Abcam, ab41489, 1:300). Alexa Fluor 647-conjugated secondary antibody (Invitrogen, Waltham, MA, USA, A32933, 1:300) was applied for 1 h at room temperature. Samples were mounted with Duolink In Situ Mounting Medium with DAPI (Sigma, DUO82040). PLA signals were imaged using a laser-scanning confocal microscope (ZEISS LSM 800, Carl Zeiss Microscopy GmbH, Jena, Germany) with a 63× objective.

### 2.13. Assessment of Mitochondrial Membrane Potential Using TMRM Staining

Mitochondrial membrane potential (MMP) was measured in embryonic DRG (eDRG) neurons using the cell-permeant dye tetramethylrhodamine methyl ester (TMRM; Thermo Fisher Scientific, I34361), which accumulates in active mitochondria, according to the manufacturer’s instructions. Briefly, cultured eDRG neurons were washed with PBS and incubated in phenol red-free Neurobasal medium (Life Technologies, Carlsbad, CA, USA, 12348-017) supplemented with 2% B-27 (Gibco, 17504044), 1% GlutaMAX, 1 μM 5-fluoro-2′-deoxyuridine (Sigma, F0503), 1 μM uridine (Sigma, U3003), 1% penicillin–streptomycin, 50 ng/mL 2.5S nerve growth factor (Envigo, BT-5017), and 100 nM TMRM reagent for 30 min at 37 °C. To visualize axons, CellMask™ Green Plasma Membrane Stain (Thermo Fisher Scientific, C37608) was added at a 1:1000 dilution during incubation. After staining, cells were washed with PBS and imaged using an EVOS™ FL Auto 2 imaging system (Thermo Fisher Scientific, AMAFD2000). TMRM-positive puncta along axons were quantified using ImageJ software (2.16.0/1.54p) and expressed as the number of puncta per 100 μm of axon length.

### 2.14. Assessment of Mitochondrial ROS Levels Using MitoSOX Staining

Mitochondrial reactive oxygen species (ROS) production in embryonic DRG (eDRG) neurons was measured using MitoSOX™ Red Mitochondrial Superoxide Indicator (Thermo Fisher Scientific, M36009) according to the manufacturer’s instructions. Briefly, cultured eDRG neurons were washed with Hank’s Balanced Salt Solution (HBSS, Thermo Fisher Scientific, 14025092) and incubated with 500 μM H_2_O_2_ in HBSS for 30 min at 37 °C to induce oxidative stress. Cells were then washed with 1× HBSS and incubated in HBSS containing 3 μM MitoSOX and 500 nM MitoTracker Green (Invitrogen, M7514) to visualize mitochondria for 30 min at 37 °C. After staining, cells were washed with 1× HBSS and imaged using an EVOS™ FL Auto 2 imaging system (Thermo Fisher Scientific, AMAFD2000). Fluorescence intensities were quantified using ImageJ software, and MitoSOX fluorescence intensity in the cell body region was normalized to the corresponding MitoTracker Green signal.

## 3. Results

### 3.1. CSDE1 Associates with the Mitochondrial Import Receptor TOM20 and Binds a Group of mRNAs Encoding Mitochondrial Proteins

Although mitochondria retain their own genome, the vast majority of mitochondrial proteins are nuclear-encoded and synthesized on cytosolic ribosomes [1,44,45,46]. Accumulating evidence supports a model of localized translation coupled to mitochondrial import, in which the synthesis of these proteins is spatially coordinated with their targeting to the mitochondrial surface and subsequent import [12,14,44,47,48,49,50,51,52]. Within this translation-translocation coupling framework, the translocase of the outer mitochondrial membrane (TOM) complex, including the import receptor TOM20, plays a central role in recognizing nuclear-encoded mitochondrial proteins and initiating their import [3,7,8,9,12,13,44,47,53,54,55,56,57,58].

Given the pivotal role of TOM20 in recognizing precursor proteins at the mitochondrial surface, we hypothesized that TOM20 might function not only as an import receptor but also as part of a molecular platform that links nuclear-encoded mitochondrial mRNAs with the mitochondrial import interface. To test this hypothesis and explore the potential interface between cytosolic translation and mitochondrial import, we employed TOM20 as a bait protein to identify interacting RNA-binding proteins (RBPs) that could mediate this regulatory crosstalk [10].

**Figure 1:**
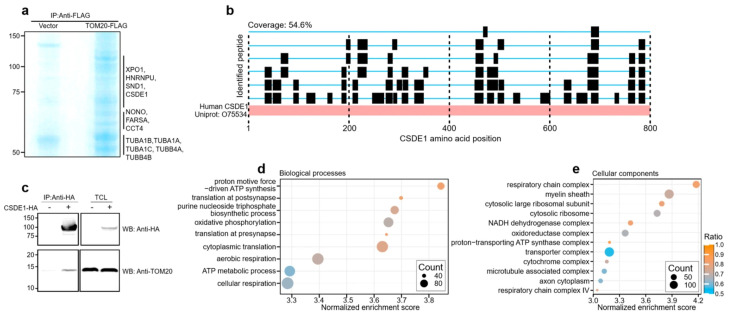
CSDE1 associates with the mitochondrial import receptor TOM20 and mitochondrial protein-encoding transcripts. (**a**) Identification of TOM20-associated proteins. HEK293T cells overexpressing FLAG-tagged TOM20 were subjected to immunoprecipitation (IP) using an anti-FLAG antibody. Proteins were separated by SDS-PAGE and visualized by Coomassie Blue staining. Proteins identified by mass spectrometry, including CSDE1, are listed on the right. (**b**) Mass spectrometric peptide coverage of CSDE1. Black bars indicate the positions of unique peptides identified by mass spectrometry mapped onto the CSDE1 amino acid sequence (54.6% sequence coverage). (**c**) Reciprocal co-immunoprecipitation of CSDE1 and TOM20. Lysates from HEK293T cells expressing HA-tagged CSDE1 were immunoprecipitated with an anti-HA antibody. Co-immunoprecipitated endogenous TOM20 was detected by Western blotting (WB). TCL, total cell lysate. (**d**,**e**) Gene Set Enrichment Analysis (GSEA) of CSDE1-associated transcripts. GSEA was performed using the CSDE1 RIP-seq ranked gene list derived from naïve mouse L4/5 DRG tissue (GSE301321). The dot plots show representative enriched Gene Ontology (GO) categories after redundancy reduction in (**d**) Biological Process (BP) and (**e**) Cellular Component (CC). The *x*-axis indicates normalized enrichment score (NES), dot size represents GeneCount (the number of genes in the core enrichment set), and color indicates GeneRatio (GeneCount/setSize). Full results are provided in Table S2.

FLAG-tagged TOM20 was overexpressed in HEK293T cells and immunoprecipitated using an anti-FLAG antibody, followed by SDS-PAGE and Coomassie staining (Figure 1a). Mass spectrometric analysis identified several proteins involved in RNA metabolism and translation as TOM20-associated factors. These include canonical RNA-binding proteins (CSDE1, HNRNPU, SND1, NONO), a tRNA synthetase (FARSA), and factors involved in nucleocytoplasmic transport (XPO1) and protein folding (CCT4) (Figure 1a). Among these candidates, CSDE1 was identified with high confidence, exhibiting a high sequence coverage (54.6%) with a substantial number of unique peptides mapping across the CSDE1 protein sequence (Figure 1b, Table S1). The full list of CSDE1-derived peptides identified in TOM20 immunoprecipitates, together with peptide confidence, spectral count, intensity, and mapped positions within the CSDE1 sequence, is provided in Table S1.

To further corroborate the physical interaction between CSDE1 and TOM20, we performed a reciprocal co-immunoprecipitation assay. CSDE1-HA was overexpressed in HEK293T cells and immunoprecipitated with an anti-HA antibody. Immunoblot analysis confirmed the specific enrichment of endogenous TOM20 in the CSDE1-HA precipitates, supporting a robust association between these two proteins (Figure 1c).

Next, to determine whether CSDE1 targets specific subsets of mRNAs linked to mitochondrial physiology, we analyzed CSDE1 RIP-seq datasets derived from uninjured naïve mouse L4/5 dorsal root ganglion (DRG) tissues in our previous work (GSE301321). Gene Set Enrichment Analysis (GSEA) demonstrated a significant enrichment of nuclear-encoded mitochondrial mRNAs among the CSDE1-bound transcripts in both Biological Process (BP) and Cellular Component (CC) categories (Figure 1d,e) [59]. Notably, Gene Ontology (GO) terms associated with mitochondrial energetics and biogenesis were highly enriched; top biological processes included ‘oxidative phosphorylation’ and ‘aerobic respiration’ (Figure 1d), and cellular components prominently featured ‘respiratory chain complex’, ‘NADH dehydrogenase complex’, ‘proton-transporting ATP synthase complex’, and ‘cytosolic ribosome’ (Figure 1e, Table S2). The full redundancy-reduced GSEA output, including enrichment statistics and core enrichment gene lists for BP and CC categories, is provided in Table S2.

Collectively, these findings identify CSDE1 as an interactor of TOM20 and reveal its preference for binding mRNAs encoding a subset of mitochondrial proteins. This suggests a potential mechanism where CSDE1 coordinates the expression of nuclear-encoded mitochondrial proteins with their subsequent import at the mitochondrial surface [10,16,17].

### 3.2. Characterization of the CSDE1-TOM20 Interaction and Mitochondrial Localization of CSDE1

To identify the region of CSDE1 responsible for the interaction with TOM20, we generated a series of HA-tagged CSDE1 truncation mutants based on its structural organization, which contains nine cold shock domains (CSDs). While the majority of these domains function as canonical RNA-binding modules, specific domains are structurally and functionally divergent [20,21,22,60]. Rather than being non-functional, these divergent CSDs are increasingly recognized for their roles in mediating protein–protein interactions and facilitating the assembly of multiprotein complexes, distinct from direct RNA binding [20,61].

**Figure 2:**
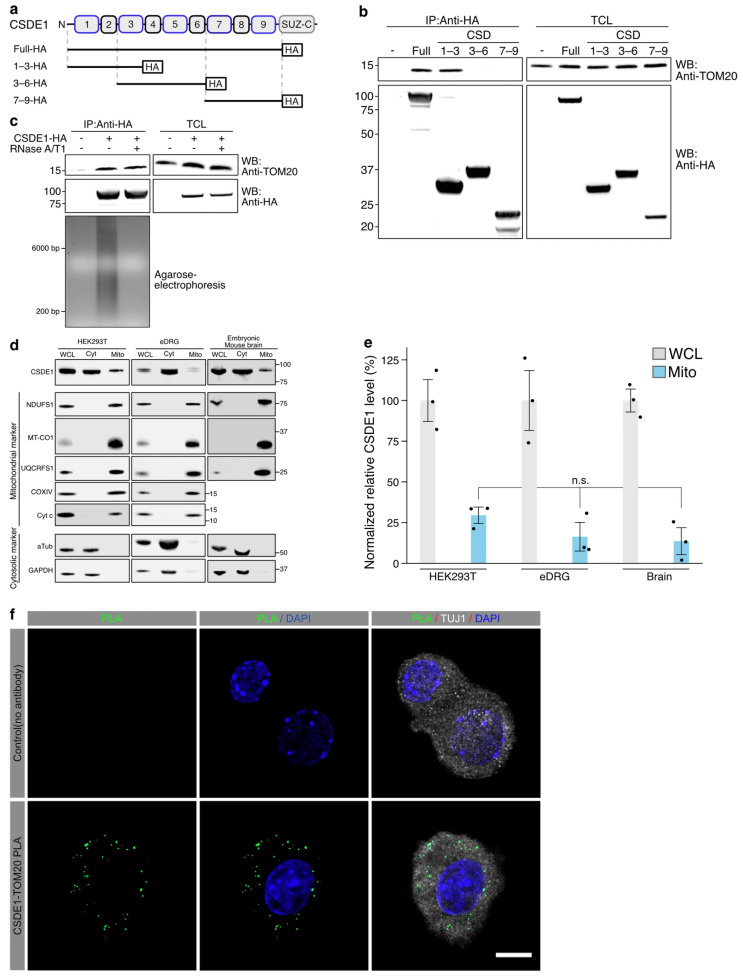
Characterization of the CSDE1-TOM20 association and mitochondrial localization of CSDE1. (**a**) Schematic representation of full-length CSDE1 and the HA-tagged truncation mutants used to map the TOM20-associated region. The constructs encompass the N-terminal (CSD 1–3), middle (CSD 3–6), and C-terminal (CSD 7–9) cold shock domains. (**b**) Domain mapping of the CSDE1-TOM20 association. HEK293T cells expressing the indicated HA-tagged CSDE1 fragments were subjected to immunoprecipitation (IP) with an anti-HA antibody. Co-immunoprecipitated endogenous TOM20 was analyzed by Western blotting. TCL, total cell lysate. (**c**) RNA-independent association between CSDE1 and TOM20. Co-immunoprecipitation was performed in HEK293T cell lysates treated with or without RNase A/T1. Retention of the CSDE1-TOM20 association was assessed by Western blotting (top), and RNA degradation was confirmed by agarose gel electrophoresis (bottom). (**d**) Subcellular fractionation analysis of endogenous CSDE1. Cytosolic (Cyt) and mitochondrial (Mito) fractions were isolated from HEK293T cells, embryonic dorsal root ganglia (eDRG), and embryonic mouse whole brains. Immunoblotting was performed for CSDE1 and markers for mitochondria (NDUFS1, MT-CO1, UQCRFS1, COXIV, Cyt c) and the cytosol (α-Tubulin, GAPDH). WCL, whole-cell lysate. (**e**) Quantification of mitochondrial-fraction CSDE1 levels. The proportion of CSDE1 detected in the mitochondrial fraction relative to the whole-cell lysate was quantified based on the results shown in (**d**). Data are presented as mean ± SD (n.s., not significant). (**f**) In situ visualization of the CSDE1–TOM20 association. Proximity ligation assay (PLA) shows endogenous proximity between CSDE1 and TOM20 (green puncta). Cells were counterstained with DAPI (nuclei, blue) and TUJ1 (neuronal marker, gray). A negative control without primary antibodies is shown to confirm signal specificity (top panels). Scale bar, 10 µm.

We constructed fragments encompassing the N-terminal (CSD 1–3), middle (CSD 3–6), and C-terminal (CSD 7–9) regions (Figure 2a). Co-immunoprecipitation assays in HEK293T cells revealed that endogenous TOM20 strongly associated with the full-length CSDE1 and the N-terminal fragment (CSD 1–3). In contrast, the CSD 3–6 and CSD 7–9 fragments failed to pull down TOM20 (Figure 2b). This result indicates that the N-terminal region of CSDE1, containing the first three cold shock domains, is necessary and sufficient for its interaction with TOM20.

Given that CSDE1 is a well-known RNA-binding protein, we next investigated whether the CSDE1-TOM20 interaction is mediated by RNA bridging. We performed co-immunoprecipitation in the presence of RNase A/T1 to digest cellular RNAs. Immunoblot analysis showed that the interaction between CSDE1 and TOM20 remained intact even after RNase treatment, comparable to the untreated control (Figure 2c). The analysis of gel electrophoresis of the immunoprecipitates confirmed the complete degradation of RNA in the protein complexes (Figure 2c). This demonstrates that the association between CSDE1 and TOM20 is a direct or protein-complex-mediated interaction rather than an RNA-bridged indirect association.

To validate the mitochondrial association of endogenous CSDE1 in a physiological context, we performed subcellular fractionation assays across different biological systems, using HEK293T cells, embryonic dorsal root ganglia (eDRG), and embryonic mouse brain tissues (Figure 2d). The purity of the cytosolic and mitochondrial fractions was confirmed using specific markers GAPDH and α-Tubulin for the cytosol and NDUFS1, MT-CO1, UQCRFS1, COXIV, and cytochrome c (Cyt c) for mitochondria. Consistent with the interaction data, a substantial population of endogenous CSDE1 was co-purified within the mitochondrial fraction in all tested samples, distinct from the cytosolic pool (Figure 2d). Quantification of the relative protein levels revealed that approximately 15–27% of the total cellular CSDE1 is localized to the mitochondrial fraction (Figure 2e). The amount of CSDE1 detected in the mitochondrial fraction was calculated relative to the corresponding whole-cell lysate signal, whereas the cytosolic and mitochondrial marker proteins were used to assess fraction purity rather than as normalization controls for this comparison.

To further confirm the endogenous CSDE1-TOM20 association in situ, we performed a proximity ligation assay (PLA) in neurons (Figure 2f). In the negative control condition (no primary antibody), PLA signals were absent, indicating low background. In contrast, robust punctate PLA signals were detected with CSDE1 and TOM20 antibodies, demonstrating close spatial proximity between endogenous CSDE1 and TOM20 in cultured embryonic DRG neurons. Co-staining with the neuronal marker TUJ1 and DAPI further verified that these PLA puncta were present in neurons, supporting the physiological relevance of the CSDE1-TOM20 interaction in a neuronal context (Figure 2f). Collectively, our results demonstrate that a specific subset of CSDE1 binds to TOM20 and localizes to mitochondria, establishing a distinct mitochondrial pool in both cell lines and neuronal tissues.

### 3.3. CSDE1 Contributes to the Enrichment of Nuclear-Encoded Mitochondrial mRNAs in the Mitochondrial Fraction

**Figure 3:**
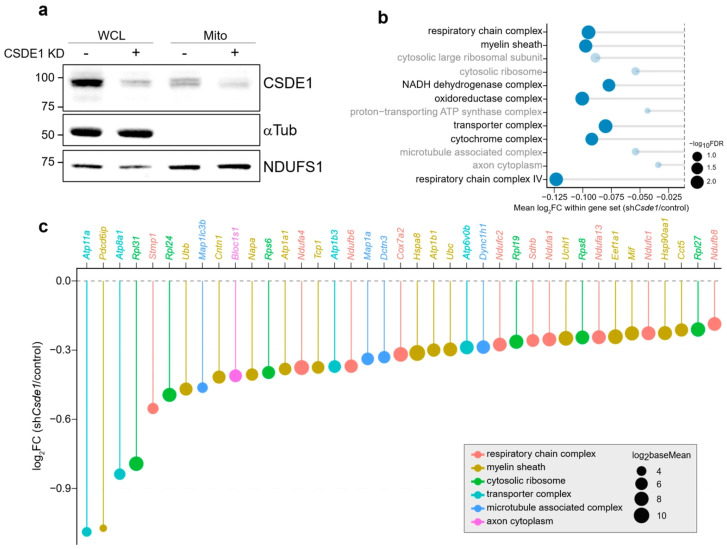
CSDE1 depletion reduces the mitochondrial-fraction abundance of nuclear-encoded mitochondrial mRNAs. (**a**) Validation of CSDE1 knockdown and subcellular fractionation efficiency. Immunoblot analysis of whole-cell lysates (WCL) and mitochondrial fractions (Mito) from control and CSDE1-knockdown (CSDE1 KD) eDRG neurons. α-Tubulin (αTub) and NDUFS1 were used as markers for the cytosolic and mitochondrial fractions, respectively. (**b**) Competitive gene set analysis of transcripts reduced in the mitochondrial fraction upon CSDE1 depletion. The dot plot displays representative Cellular Component gene sets that were significantly downregulated in the mitochondrial fraction of CSDE1-knockdown neurons compared with controls. The *x*-axis indicates mean log_2_ fold change (FC), and dot size represents −log10 FDR. (**c**) Differential expression of representative nuclear-encoded mitochondrial mRNAs. The lollipop chart shows transcripts significantly reduced in the mitochondrial fraction following CSDE1 depletion. Genes are color-coded according to functional category (e.g., respiratory chain complex, transporter complex), and dot size corresponds to mean expression level (log_2_ baseMean).

We next hypothesized that CSDE1 functionally mediates the recruitment or retention of its target mRNAs to the mitochondrial compartment. To test this, we performed subcellular fractionation coupled with RNA sequencing (Mito-RNA-seq) in control and CSDE1-depleted (sh*Csde1*) eDRG neurons. First, the efficiency of CSDE1 knockdown and the purity of the mitochondrial fraction were validated by immunoblot analysis (Figure 3a). CSDE1 protein levels were markedly reduced in both whole-cell lysates (WCL) and the mitochondrial fraction (Mito) upon shRNA delivery via lentiviral infection, while the cytosolic marker α-Tubulin and the mitochondrial marker NDUFS1 confirmed successful fractionation (Figure 3a).

We then analyzed the transcriptome associated with the mitochondrial fraction using the limma package (3.64.1) [62]. To determine whether the loss of CSDE1 specifically affects the mitochondrial localization of its RNA targets identified in Figure 1, we performed CAMERA, a competitive gene set test that accounts for inter-gene correlation [63]. We interrogated the specific Gene Ontology (GO) categories that were previously identified as highly enriched in the CSDE1 RIP-seq dataset from naïve L4/5 DRG (as shown in Figure 1e). This analysis revealed a significant depletion of these CSDE1-target gene sets from the mitochondrial fraction in CSDE1-knockdown neurons. Specifically, gene sets associated with the ‘respiratory chain complex IV’, ‘NADH dehydrogenase complex’, ‘oxidoreductase complex’, and ‘proton-transporting ATP synthase complex’ showed significant negative mean log2 fold changes (FC) (Figure 3b, Table S3). The complete CAMERA output for all tested gene sets, including mean log_2_ fold change and enrichment statistics, is provided in Table S3.

These results indicate that CSDE1 loss reduces the mitochondrial-fraction abundance of these CSDE1-associated mRNAs. Interestingly, transcripts encoding cytosolic ribosomal subunits were concordantly reduced in the mitochondrial fraction, suggesting a potential defect in the assembly of the translation machinery at the mitochondrial surface (Figure 3b). In addition, analysis of individual transcripts confirmed the widespread downregulation of specific nuclear-encoded mitochondrial mRNAs. Key subunits of the electron transport chain, such as Ndufa4, Ndufa1, Ndufc1, Ndufb8 (Complex I), and Cox7a2 (Complex IV), along with mitochondrial transporters (Atp11a, Atp8a1) and ribosomal proteins (Rpl31, Rpl24, Rps6), were among the top downregulated genes in the mitochondrial fraction of CSDE1-depleted cells (Figure 3c). Collectively, these data indicate that CSDE1 contributes to the mitochondrial-fraction enrichment of a specific subset of nuclear-encoded mitochondrial mRNAs, supporting a model in which CSDE1 participates in mitochondria-associated post-transcriptional regulation.

### 3.4. CSDE1 Depletion Reduces Mitochondrial Protein Abundance and TMRM-Positive Mitochondrial Puncta Density in Sensory Neurons

**Figure 4:**
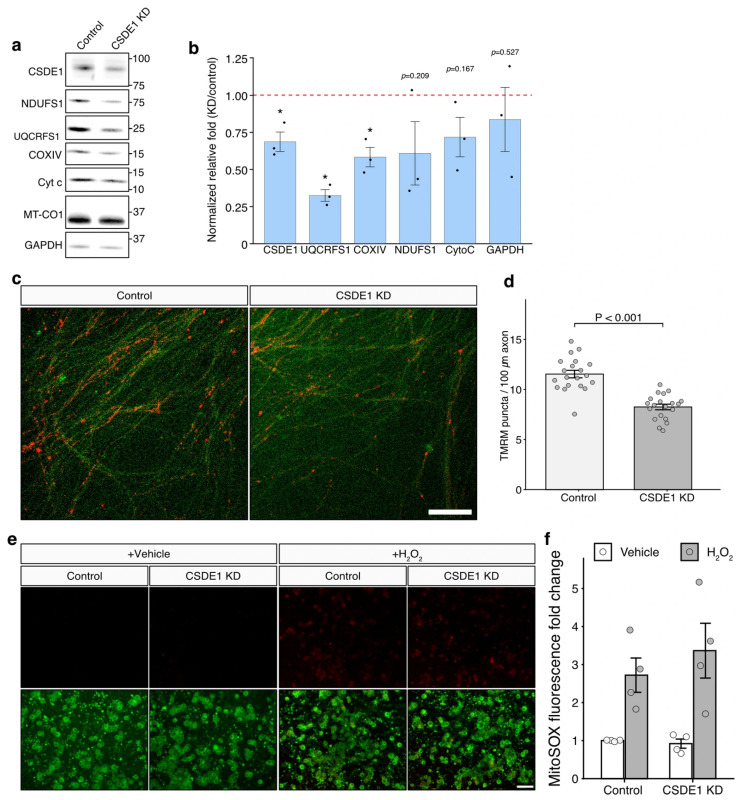
CSDE1 depletion reduces mitochondrial protein abundance and TMRM-positive mitochondrial puncta density in sensory neurons. (**a**) Western blot analysis of representative mitochondrial proteins in mitochondrial fractions from control and CSDE1-knockdown eDRG neurons. Proteins encoded by nuclear genes associated with mitochondrial pathways were examined to determine whether reduced mitochondrial-fraction mRNA abundance upon CSDE1 depletion was accompanied by decreased protein abundance. (**b**) Quantification of the immunoblot signals shown in (**a**). Protein levels were normalized to the mitochondrial loading control (MT-CO1) and expressed relative to control samples (mean ± S.E.M., *n* = 3). (**c**) Representative TMRM fluorescence images of control and CSDE1-knockdown sensory neurons. TMRM-positive mitochondrial puncta were visualized along neuronal processes. Scale bar, 100 μm. (**d**) Quantification of TMRM-positive puncta density per unit neurite length. CSDE1 depletion significantly reduced the number of TMRM-positive puncta per unit length by 28.5% compared with control neurons. Data are presented as mean ± S.E.M. (**e**) Representative fluorescence images of MitoSOX staining in control and CSDE1-knockdown sensory neurons treated with vehicle or H_2_O_2_. MitoSOX was used to assess mitochondrial superoxide-associated fluorescence under basal and oxidative challenge conditions. (**f**) Quantification of MitoSOX fluorescence intensity in vehicle- and H_2_O_2_-treated control and CSDE1-knockdown neurons. Data are presented as mean ± S.E.M. * *p* < 0.05 by Student’s *t*-test. The red dashed line indicates a fold change of 1 on the y-axis. Scale bars: 100 μm in Figure (**c**) and 200 μm in Figure (**e**).

To determine whether the reduced mitochondrial localization of these nuclear-encoded mitochondrial transcripts upon CSDE1 depletion translates into altered protein abundance, we next examined the levels of selected mitochondrial proteins in the mitochondrial fraction. Mitochondrial fractions were isolated from control and CSDE1 knockdown eDRG neurons and subjected to immunoblot analysis for representative proteins encoded by the downregulated transcripts. Consistent with the RNA-seq results, several mitochondrial proteins showed a reduction in the mitochondrial fraction upon CSDE1 depletion (Figure 4a). Quantification of the immunoblot signals revealed a consistent decrease in protein abundance relative to control samples, indicating that the loss of CSDE1 not only disrupts the localization of mitochondrial mRNAs but also compromises the accumulation of their encoded proteins at the mitochondrial compartment (Figure 4b). Together, these results support a model in which CSDE1-dependent enrichment of nuclear-encoded mitochondrial mRNAs contributes to the accumulation of their encoded proteins at the mitochondrial compartment.

We next asked whether reduced mitochondrial protein abundance was accompanied by changes in mitochondrial labeling in sensory neurons. We stained control and CSDE1-depleted sensory neurons with TMRM, a membrane-potential-dependent mitochondrial dye, and quantified TMRM-positive mitochondrial puncta along neurites. Compared with control neurons, CSDE1-depleted neurons showed a visible reduction in TMRM-positive puncta along neuronal processes (Figure 4c). Quantitative analysis revealed that CSDE1 depletion reduced the number of TMRM-positive puncta per unit neurite length by 28.5% relative to control neurons (Figure 4d). These data suggest that CSDE1 depletion is associated with reduced abundance or maintenance of polarized mitochondrial puncta in sensory neurites.

Because impaired mitochondrial protein accumulation and reduced TMRM-positive puncta density could potentially influence mitochondrial oxidative stress, we further assessed mitochondrial superoxide-associated fluorescence using MitoSOX under basal and oxidative challenge conditions. Control and CSDE1-depleted sensory neurons were treated with either vehicle or H_2_O_2_ and then stained with MitoSOX. H_2_O_2_ treatment increased MitoSOX fluorescence compared with vehicle-treated conditions, confirming the induction of oxidative stress-associated mitochondrial superoxide signal (Figure 4e). However, CSDE1 depletion did not significantly alter MitoSOX fluorescence compared with control neurons under either vehicle- or H_2_O_2_-treated conditions (Figure 4f). Thus, while CSDE1 depletion reduced mitochondrial protein abundance and TMRM-positive mitochondrial puncta density, it did not detectably enhance mitochondrial superoxide-associated fluorescence under the conditions tested. Together, these findings suggest that CSDE1 contributes to the maintenance of mitochondrial protein accumulation and polarized mitochondrial puncta in sensory neurons, without producing a significant change in MitoSOX-detectable mitochondrial superoxide levels.

## 4. Discussion

Mitochondrial proteostasis depends on the coordinated production, targeting, import, and assembly of a predominantly nuclear-encoded proteome [1,5,6,44]. This coordination is especially important in neurons, where mitochondrial function must support high and spatially heterogeneous energetic demands across extended cellular compartments [64,65,66,67].

In this study, we identify the RNA-binding protein CSDE1 (UNR) as a TOM20-associated post-transcriptional factor linked to mitochondrial protein-encoding mRNAs in sensory neurons. Our data show that CSDE1 is associated with a broad cohort of nuclear-encoded mitochondrial mRNAs in sensory neurons, with enrichment for transcripts encoding inner membrane and matrix proteins, including pathways related to the respiratory chain and respirasome. Precise regulation of these pathways is important because the electron transport chain (ETC) is central to cellular ATP production and is closely linked to mitochondrial metabolic state. MitoCarta-based compartment annotation provides an important context for this bias because inner membrane/matrix proteins constitute a substantial fraction of import-intensive and assembly-constrained mitochondrial gene products [68,69]. In addition, CSDE1 depletion reduced the mitochondrial-fraction abundance of representative nuclear-encoded mitochondrial proteins and decreased TMRM-positive mitochondrial puncta density in sensory neurons, whereas MitoSOX-detectable mitochondrial superoxide signals were not significantly altered by CSDE1 depletion under either basal or H_2_O_2_-treated conditions. A fraction of CSDE1 is partitioned with mitochondria and associated with the outer-membrane import receptor TOM20 in an RNA-independent manner. Together, these findings support a model in which CSDE1 is associated with selective mitochondrial mRNA regulation and early steps of mitochondrial protein biogenesis, with measurable consequences for mitochondrial protein accumulation and TMRM-positive mitochondrial organization in sensory neurons.

### 4.1. CSDE1 Is Associated with a Mitochondria-Related mRNA Cohort

RIP-seq enrichment demonstrates that CSDE1 binds a large class of mRNAs encoding mitochondrial proteins, extending CSDE1 biology beyond previously recognized transcript cohorts [22,70,71,72]. The functional bias of this cohort is notable, as many CSDE1-bound transcripts encode proteins destined for the inner membrane or matrix, and gene set enrichment highlights respirasome-related categories. These proteins are among the most biogenesis-demanding classes because they must be synthesized in the cytosol, maintained in an import-competent state, translocated through TOM and TIM machineries, and then inserted and assembled into multi-subunit complexes with strict stoichiometric constraints [1,5,6,44]. Thus, the CSDE1-associated mitochondrial transcript set is enriched for gene products whose steady-state abundance may be particularly sensitive to inefficiencies at translation, import, or assembly.

This concept aligns with a broader view of CSDE1 as a context-dependent organizer of post-transcriptional gene expression programs [22,72,73,74]. CSDE1 has been implicated in shaping cell-state transitions and disease phenotypes through translational control and mRNA fate regulation, and its versatility and partner-dependent output [60,75]. Our findings suggest that, in sensory neurons, one prominent CSDE1-associated program is related to mitochondrial transcript and protein regulation [23].

### 4.2. RNA-Independent CSDE1-TOM20 Association Is Consistent with a Link to the Mitochondrial Import Machinery

CSDE1 associates with TOM20 in an RNA-independent manner, and this interaction maps to the N-terminal region encompassing CSD1–3. TOM20 is a core receptor of the TOM complex, recognizing N-terminal targeting presequences and coordinating early steps of mitochondrial protein import [1,6,8,12,44,57,76]. Structural studies define a presequence-binding groove in TOM20 that accommodates amphipathic targeting helices [55], and TOM20 is proposed to recognize diverse presequences through a dynamic equilibrium among multiple bound states [58]. These properties place TOM20 at a strategic and versatile first-contact position for incoming precursors.

Import receptors also contribute to proteostasis at the mitochondrial surface. In particular, TOM20 and TOM22 have been shown to display chaperone-like activity toward unfolded polypeptides, suppressing aggregation and thereby helping maintain import competence at the outer membrane [57]. This receptor-proximal staging function is conceptually well matched to an RBP-based mechanism. If CSDE1 associates with mitochondria-destined mRNAs and with TOM20, it could help enrich selected transcripts near the import gate and thereby support mitochondria-proximal post-transcriptional regulation.

Our finding that CSDE1-TOM20 binding persists after RNase A/T1 treatment argues that CSDE1 does not associate with TOM20 merely through RNA bridging. This expands the classical view of mitochondrial import in which cytosolic targeting factors, chaperones, and receptors are linked primarily through precursor proteins themselves. It also complements the long-standing recognition that the outer membrane import apparatus is modular and cooperative, with receptor overlap and translocase cooperation shaping substrate handling [7,8,54,56,77]. In this framework, CSDE1 may represent an additional regulatory layer linking selective RNA association to the TOM20-proximal import interface [10,16,17].

### 4.3. Placing CSDE1 Within Mitochondrial mRNA Localization and Translation-Associated Import Frameworks

Translation and mitochondrial import are often presented as sequential steps, yet a growing body of work supports spatial and temporal coupling between where a protein is synthesized and where it is imported, at least for a defined subset of mitochondrial substrates [78]. A foundational observation dating back to classic yeast work showed that cytosolic ribosomes can physically associate with the mitochondrial outer membrane, suggesting that translation may occur in proximity to the organelle surface [49].

Subsequent synthesis across systems has consolidated this concept by explicitly reviewing evidence for localized translation on the cytosolic side of the outer mitochondrial membrane and proposing conserved factors and pathways that support it [79]. In mammalian cells, transcriptome-scale proximity mapping approaches have provided modern support for this spatial organization by identifying distinct pathways of mRNA localization to mitochondria, including outer-membrane-proximal RNA populations [80]. Consistent with these datasets, recent reviews emphasize that many nuclear-encoded mitochondrial mRNAs can be enriched at the outer mitochondrial membrane in pre- or cotranslational states and can be locally translated to feed cotranslational import. Beyond bulk localization, mechanistic rules for cotranslational mitochondrial import have been quantified in human cells using selective ribosome profiling, supporting the idea that a substantial fraction of mitochondrial proteins can enter mitochondria during translation [16]. Complementary proximity-specific ribosome profiling approaches further reinforce that localized translation is a broad strategy for spatiotemporal control of gene expression, including translation occurring in mitochondria-proximal space [15].

Importantly, neuron-focused studies have strengthened the physiological relevance of these concepts by showing that mitochondria can serve as platforms for local translation in extended cellular architectures where distal compartments cannot rely solely on somatic protein supply [19]. In particular, neuronal mitochondria can cotransport specific mRNAs (e.g., Pink1) such that local translation in distal neurites becomes necessary to sustain compartmentalized mitochondrial quality control pathways like mitophagy.

Structural analyses of neurons reveal mitochondria-enriched axonal branch points with concentrated ribosome populations, supporting a model in which mitochondria-adjacent regions contribute to compartmentalized protein synthesis during neuronal morphogenesis [81]. More recently, high-resolution imaging and cryo-electron tomography have identified ribosome-rich neuronal RNA granules positioned around mitochondria that function as sites of local translation, providing direct evidence for mitochondria-adjacent protein synthesis microenvironments in neurons [18]. Finally, neuron and disease-relevant pathways can actively regulate this mitochondria-proximal translation layer, as exemplified by PINK1/Parkin-mediated control of localized translation of nuclear-encoded respiratory chain component mRNAs at the mitochondrial outer membrane [82].

Within this landscape, our data suggest that CSDE1 may represent a plausible molecular link between selective mitochondrial mRNAs and the outer membrane import machinery. We propose a working model in which CSDE1 associates with a defined cohort of mitochondria-related mRNAs and, through RNA-independent association with TOM20, contributes to their enrichment in the mitochondrial fraction. Such positioning could be relevant for mitochondria-proximal regulation of transcripts encoding inner membrane/matrix OXPHOS-related proteins, which are particularly sensitive to import and assembly constraints. Notably, TOM20 itself requires proper docking and assembly within the TOM complex for full receptor function [9], emphasizing that receptor-proximal regulation can be a crucial control point in mitochondrial proteostasis.

Consistent with this model, CSDE1 depletion reduced the mitochondrial-fraction abundance of selected nuclear-encoded mitochondrial proteins whose transcripts were decreased in the mitochondrial fraction. This finding links the transcript-level changes observed by Mito-RNA-seq to altered accumulation of representative mitochondrial proteins. Moreover, CSDE1 depletion reduced the density of TMRM-positive mitochondrial puncta along sensory neurites, suggesting that impaired CSDE1-dependent mitochondrial mRNA regulation may be accompanied by altered maintenance or distribution of polarized mitochondrial structures. However, CSDE1 depletion did not significantly change MitoSOX fluorescence under either vehicle- or H_2_O_2_-treated conditions. Thus, the functional consequence detected in our assays appears more closely associated with reduced mitochondrial protein accumulation and TMRM-positive puncta density than with a detectable increase in mitochondrial superoxide levels under the conditions tested.

Mitochondrial protein import dysfunction and downstream failures in respiratory chain biogenesis contribute to a spectrum of neurological disorders, including peripheral neuropathies and Parkinson’s disease [83]. In neurons, this is particularly consequential because mitochondrial homeostasis must be maintained within an extended cellular architecture through regulated trafficking, local positioning, and renewal mechanisms [67,82]. Our observation that CSDE1 depletion reduces the abundance of selected respiratory-chain-related mRNAs and proteins in the mitochondrial fraction, together with the reduced density of TMRM-positive mitochondrial puncta, suggests that perturbations in CSDE1 function may influence mitochondria-associated gene regulation and mitochondrial organization in settings where protein import and respiratory-chain biogenesis are vulnerable.

Finally, our work highlights several important directions for future studies. Defining the signals and molecular partners that control CSDE1 recruitment to mitochondria will be an essential next step. It will be critical to determine whether neuronal activity, injury, mitochondrial stress, or specific signaling pathways dynamically modulate CSDE1 association with the mitochondrial surface. In addition, it will be important to clarify the scope and specificity of CSDE1’s mitochondrial RNA program, including whether it preferentially binds and regulates subsets of mitochondrial mRNAs that encode highly important or assembly-critical subunits. Future work should also determine whether the reduction in TMRM-positive mitochondrial puncta reflects altered mitochondrial membrane potential, mitochondrial abundance, morphology, trafficking, or local maintenance in neurites.

Several limitations of the present study should be noted. First, several interaction-focused mechanistic assays were performed in HEK293T cells for biochemical tractability, although neuronal relevance was supported by DRG RIP-seq, neuronal PLA, mitochondrial fractionation in neuronal samples, and CSDE1 knockdown analyses in sensory neurons. Second, our data do not directly resolve CSDE1 binding sites on mitochondrial transcripts or directly test localized translation at mitochondria. Third, although we assessed TMRM-positive mitochondrial puncta density and MitoSOX-detectable mitochondrial superoxide signals, these assays do not by themselves distinguish among changes in mitochondrial membrane potential, mitochondrial mass, morphology, trafficking, or local maintenance. In addition, we did not directly measure mitochondrial respiration, import efficiency, or nascent mitochondrial protein synthesis. These questions will be important to address in future studies.

In conclusion, our findings identify CSDE1 as an RNA-binding protein associated with TOM20 and mitochondrial protein-encoding transcripts in sensory neurons. By coupling selective RNA association with RNA-independent association with TOM20, CSDE1 may contribute to mitochondria-associated post-transcriptional regulation in sensory neurons. The reduction in mitochondrial-fraction mRNAs, selected mitochondrial proteins, and TMRM-positive mitochondrial puncta upon CSDE1 depletion further supports the functional relevance of this regulatory axis. This work not only expands our understanding of CSDE1’s role in neuronal RNA biology but also suggests a mechanism by which RNA-binding proteins may intersect with the mitochondrial import machinery in a neuron-relevant context.

## 5. Conclusions

Our findings identify CSDE1 as a mitochondria-associated RNA-binding protein that associates with TOM20 and mitochondrial protein-encoding transcripts in sensory neurons. CSDE1 depletion reduces the mitochondrial-fraction abundance of nuclear-encoded mitochondrial mRNAs and selected mitochondrial proteins and decreases TMRM-positive mitochondrial puncta density along sensory neurites. These results support a model in which CSDE1 contributes to mitochondria-associated post-transcriptional regulation and helps maintain mitochondrial protein accumulation and polarized mitochondrial organization in sensory neurons.

## Data Availability

The RNA-sequencing data generated in this study are publicly available in the Gene Expression Omnibus (GEO) database under accession numbers GSE301321 and GSE324285. The RNA-sequencing data generated in this study are publicly available in the Gene Expression Omnibus (GEO) database under accession numbers GSE301321 and GSE324285 (https://www.ncbi.nlm.nih.gov/geo/query/acc.cgi?acc=GSE301321; https://www.ncbi.nlm.nih.gov/geo/query/acc.cgi?acc=GSE324285; accessed on 4 May 2026).

## Supplementary Materials

The following supporting information can be downloaded at: https://www.mdpi.com/article/10.3390/antiox15050608/s1, Table S1: Mass spectrometric parameters and peptide sequences of CSDE1 identified in TOM20 immunoprecipitates; Table S2: Gene Set Enrichment Analysis (GSEA) of CSDE1-bound mRNAs identified by RIP-seq in mouse DRG tissues; Table S3: Competitive gene set testing (CAMERA) of mitochondrial-associated transcriptomes in CSDE1-knockdown neurons; Supplementary File S1: Original Western blot images.

## Abbreviations

The following abbreviations are used in this manuscript:

3′UTR	3′ untranslated region
ABC	ammonium bicarbonate
ACN	acetonitrile
AGC	automatic gain control
ATP	adenosine triphosphate
BP	biological process
BSA	bovine serum albumin
CC	cellular component
CSDE1	cold shock domain-containing E1
CSD	cold shock domain
DAPI	4′,6-diamidino-2-phenylindole
DIV	days in vitro
DRG	dorsal root ganglion
DTT	dithiothreitol
DW	deionized water
eDRG	embryonic dorsal root ganglion
ECL	enhanced chemiluminescence
EDTA	ethylenediaminetetraacetic acid
ETC	electron transport chain
FA	formic acid
FC	fold change
FDR	false discovery rate
GAPDH	glyceraldehyde 3-phosphate dehydrogenase
GO	gene ontology
GSEA	gene set enrichment analysis
HA	hemagglutinin
HCD	higher-energy collisional dissociation
HEK293T	human embryonic kidney 293T
HPLC	high-performance liquid chromatography
IAA	iodoacetamide
IP	immunoprecipitation
LC-MS/MS	liquid chromatography-tandem mass spectrometry
L4/5	lumbar 4 and 5
OXPHOS	oxidative phosphorylation
PBS	phosphate-buffered saline
PBS-T	phosphate-buffered saline containing Tween-20
PCR	polymerase chain reaction
PEI	polyethylenimine
PLA	proximity ligation assay
RBP	RNA-binding protein
RIP-seq	RNA immunoprecipitation sequencing
RNP	ribonucleoprotein
RNase	ribonuclease
ROS	reactive oxygen species
SDS	sodium dodecyl sulfate
SDS-PAGE	sodium dodecyl sulfate-polyacrylamide gel electrophoresis
shRNA	short hairpin RNA
TBS	Tris-buffered saline
TBS-T	Tris-buffered saline containing Tween-20
TCA	trichloroacetic acid
TIM	translocase of the inner mitochondrial membrane
TCL	total cell lysate
TUJ1	neuron-specific class III β-tubulin
WB	Western blot
WCL	whole-cell lysate
