# Supplementary material for: CSDE1 Associates with TOM20 and Mitochondrial Protein-Encoding mRNAs in Sensory Neurons
Source: Antioxidants (Basel). 2026 May 11;15(5):608. doi: 10.3390/antiox15050608 (PMC13203772; doi:10.3390/antiox15050608)
Supplement: Supplementary file 1 [file antioxidants-15-00608-s001.zip › TableS1.pdf]

Peptide	Conf	XCorr	Total Intensity	Count	Length	Position
KSPAAPGQSPTGSVCYERN	99.8	19451	65861756	1	19	115
KEAEDGIIAYDDCGVKL	100	34371	53477756	3	17	452
KGEVYPFGIVGMANKG	100	29611	49832015	2	16	628
RATVECVKDQFGFINYEVGDSKK	100	39165	49575456	3	23	675
RCQGVVCAMKE	99.5	24292	47876815	3	11	185
KGDLETLQPGDDVEFTIKD	100	4313	45847555	1	19	217
KVEFSISDKQ	99.6	1715	40138105	1	10	488
KIKQEILPEERM	99.9	2884	36220892	2	12	89
KVDFVIPKE	99.8	21462	31756188	1	9	288
RLKNITLDDASAPRL	100	41412	31675552	2	15	756
KEKEAEDGIIAYDDCGVKL	100	63081	28710852	2	19	450
KDVEGSTSPQIGDKV	100	28664	28664698	1	15	475
KSKVTLLEGDHVRF	99.9	43341	27234118	2	14	305
KLTIAFQAKD	100	21855	25887132	1	10	467
KLLTSYGFIQCSERQ	99.9	35504	24530112	3	15	32
KTHSVNGITEEADPTIYSGKV	100	60208	21340468	1	21	581
RLLPQGTVIFEDISIEHFEGTVTKV	100	53719	20433169	1	25	246
KDQFGFINYEVGDSKK	100	38389	18440788	1	16	682
RLFFHCSQYNGNLQDLKV	100	64868	17880031	2	18	48
KEIFFHYSEFKG	99.7	30569	17426178	2	12	207
KQRPGQQVATCVRL	99.9	33986	17316836	2	14	496
KDQFGFINYEVGDSKKL	99.9	5127	14910503	2	17	682
REMGVIAAMRD	99.1	22502	12722958	1	11	348
KELPFGDKDTKS	99.7	23546	12407289	1	12	295
RIKVDFVIPKE	99.9	25001	12370906	1	11	286
KVTLLEGDHVRF	100	28119	12186278	1	12	307
KVPSKNQNDPLPGRI	99.7	17815	11285449	2	15	273
KINFVIDNNKH	99.9	25906	11009490	1	11	157
KVGDDVEFEVSSDRRT	99.9	39486	10870716	2	16	64
RGPDNSMGFGAERK	100	33083	9179564	5	14	777
KNQNDPLPGRI	100	24463	9011780	2	11	277
KFQLCVLGQNAQTMAYNITPLRR	100	5	8918869	1	23	653
KDNFGFIETANHDKE	99.9	39319	8126417	1	15	527
KDVEGSTSPQIGDKVEFSISDKQ	99.9	5546	7645025	2	23	475
KEAFGFIERG	99.7	20098	7451675	2	10	194
KCSACNVWRV	100	2798	6554997	1	10	729
RATNIEVLSNTFQFTNEARE	100	64441	4632599	2	20	330
KGDLETLQPGDDVEFTIKDRN	100	5192	3998460	2	21	217
RPGQQVATCVRL	100	32191	2524176	1	12	498
KNITLDDASAPRL	100	25287	2429697	2	13	758
