# Supplementary figures and images for: CSDE1 Associates with TOM20 and Mitochondrial Protein-Encoding mRNAs in Sensory Neurons
Source: Antioxidants (Basel). 2026 May 11;15(5):608. doi: 10.3390/antiox15050608 (PMC13203772; doi:10.3390/antiox15050608)

Fig 1c

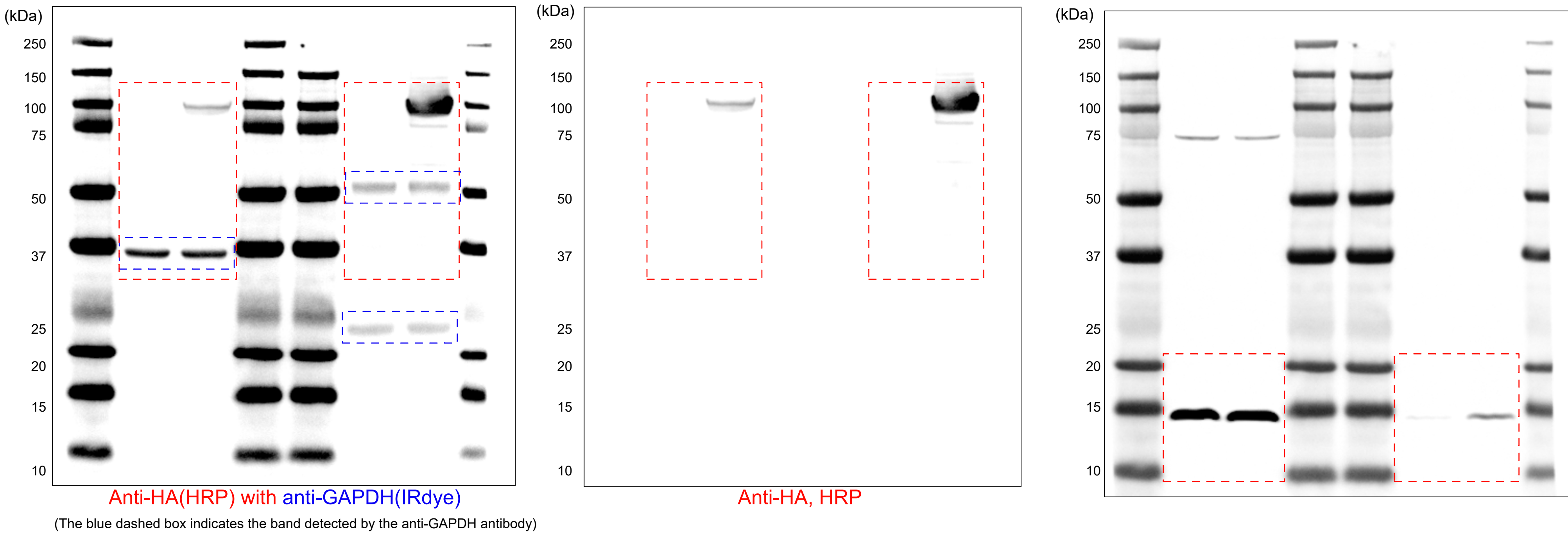

Fig 2b

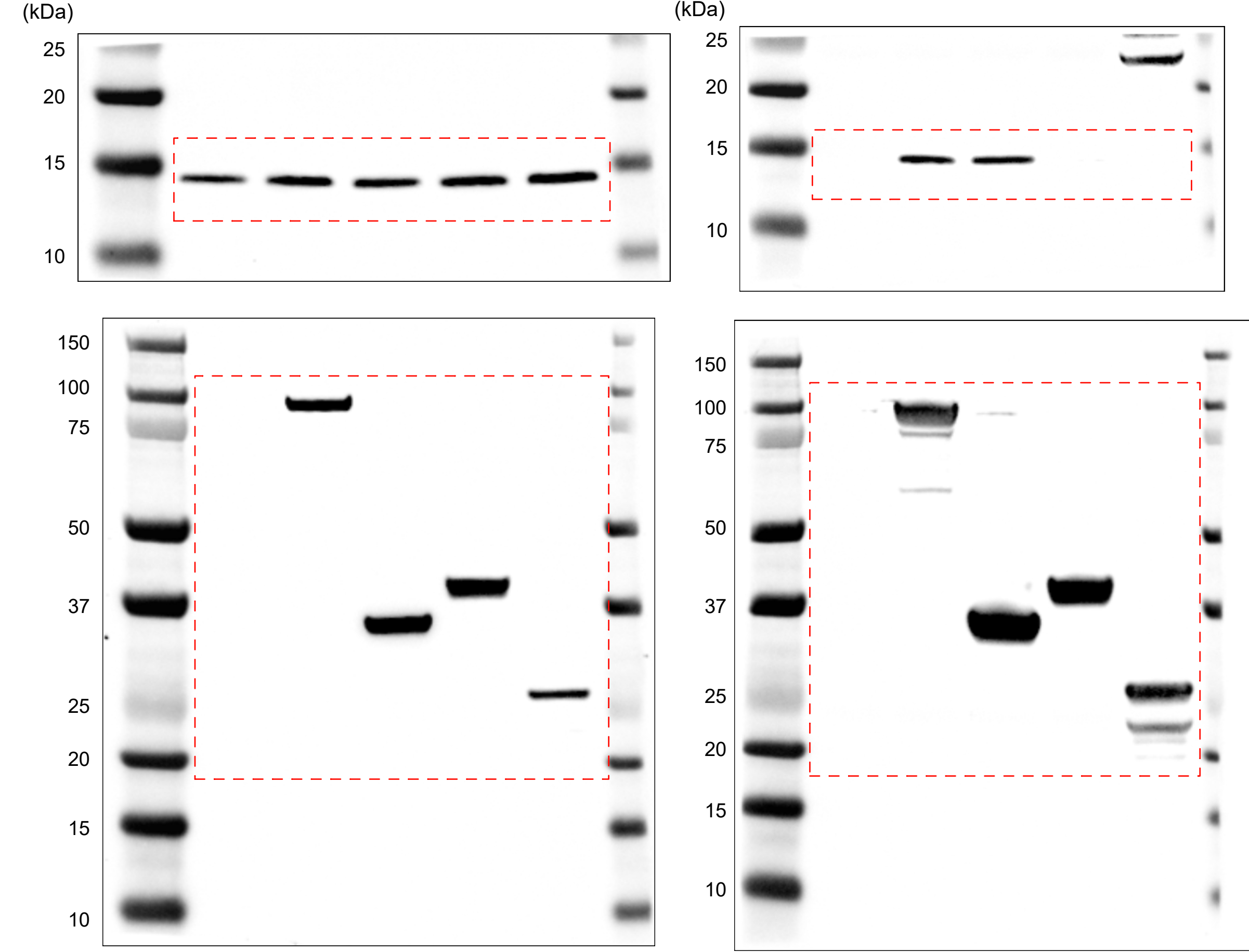

Fig 2c

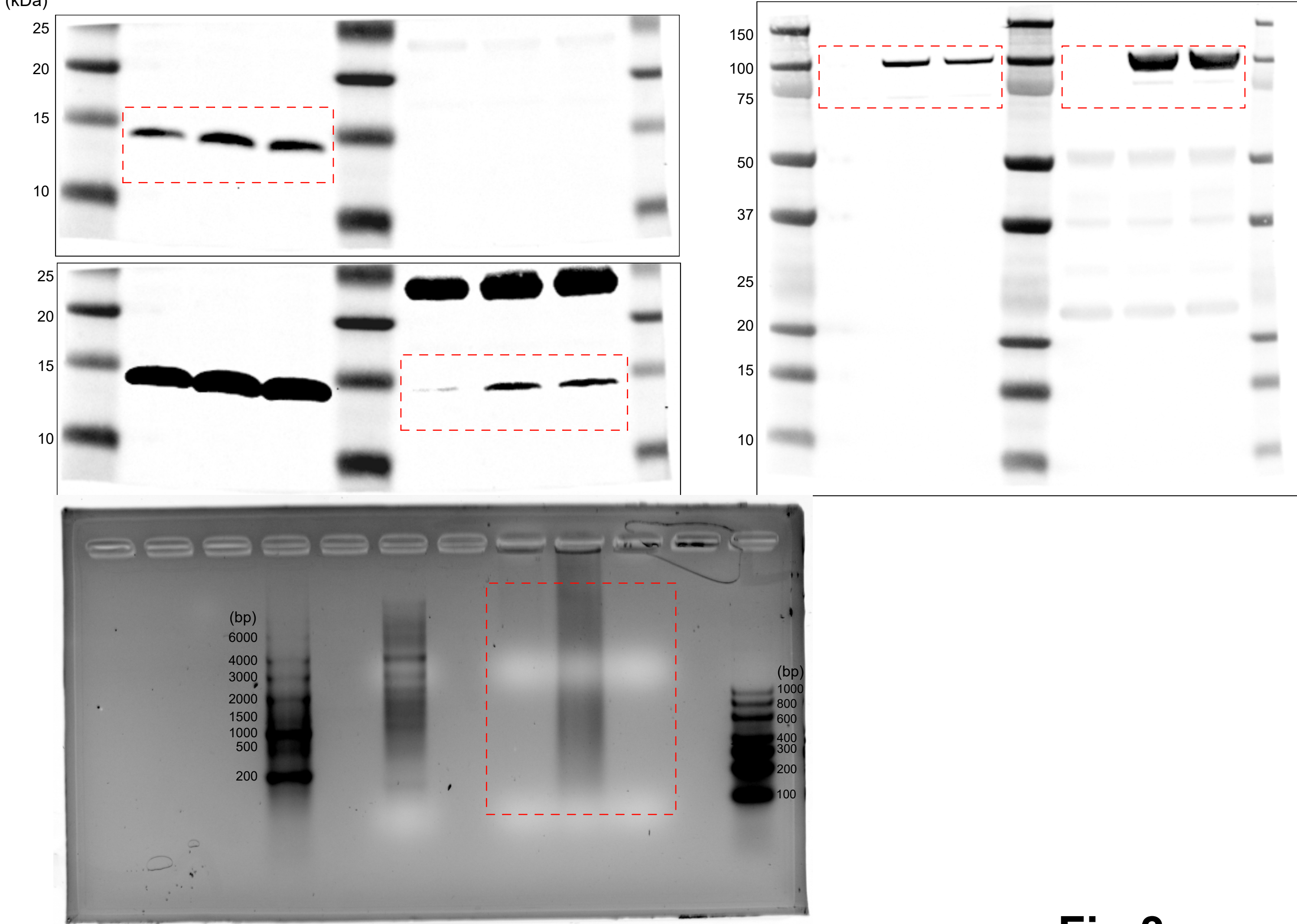

Fig 2d

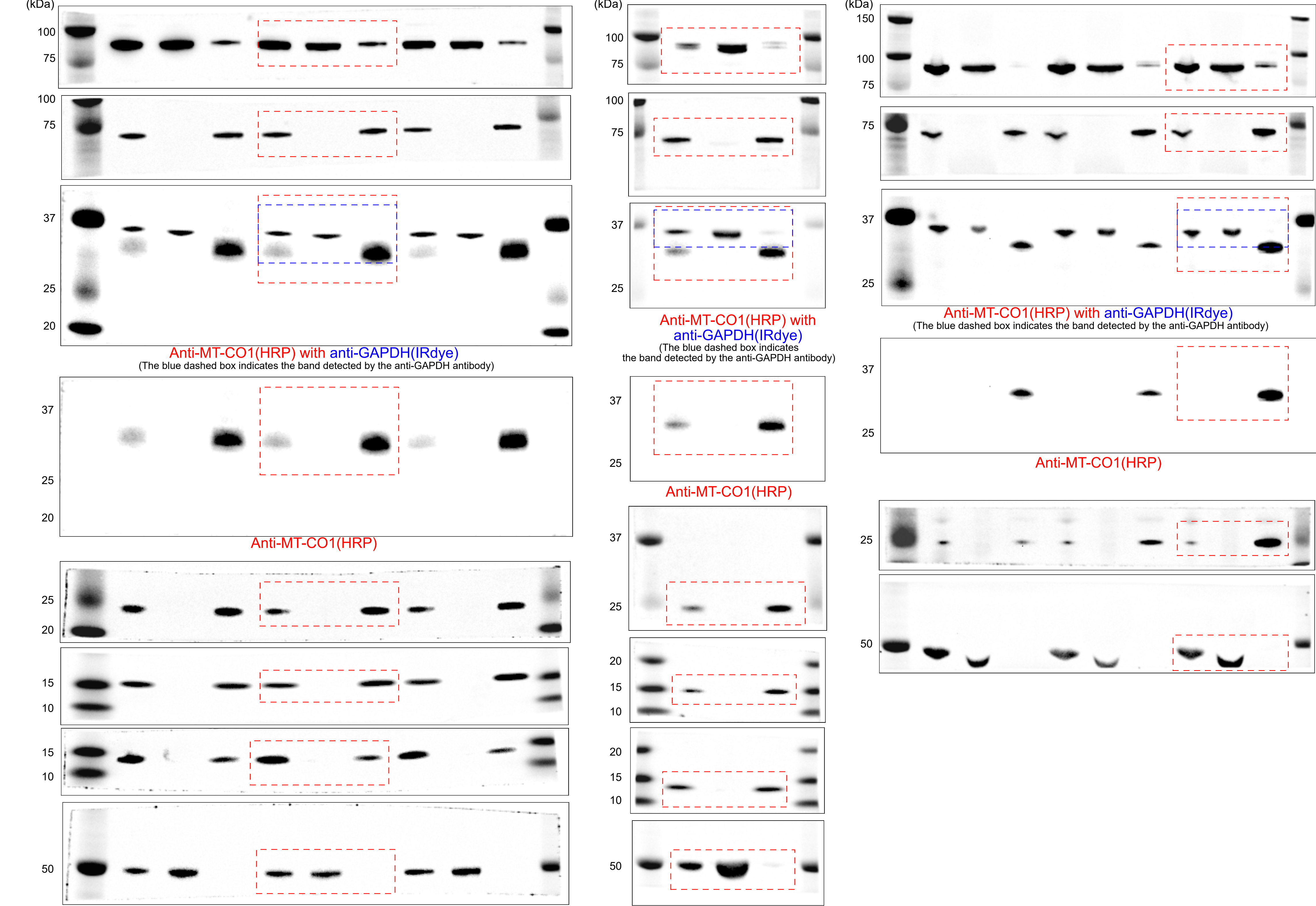

Fig 3a

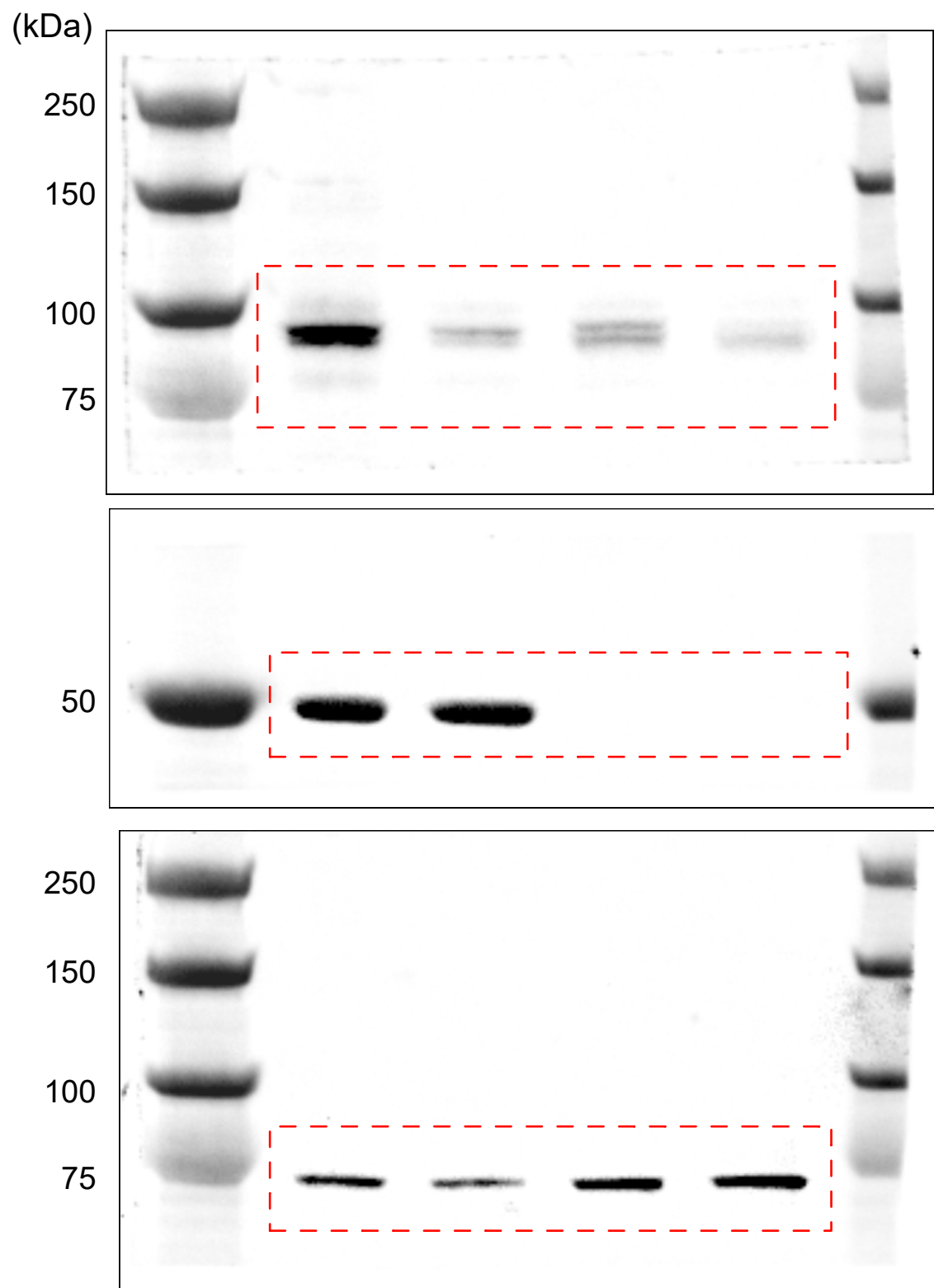

Fig 3d

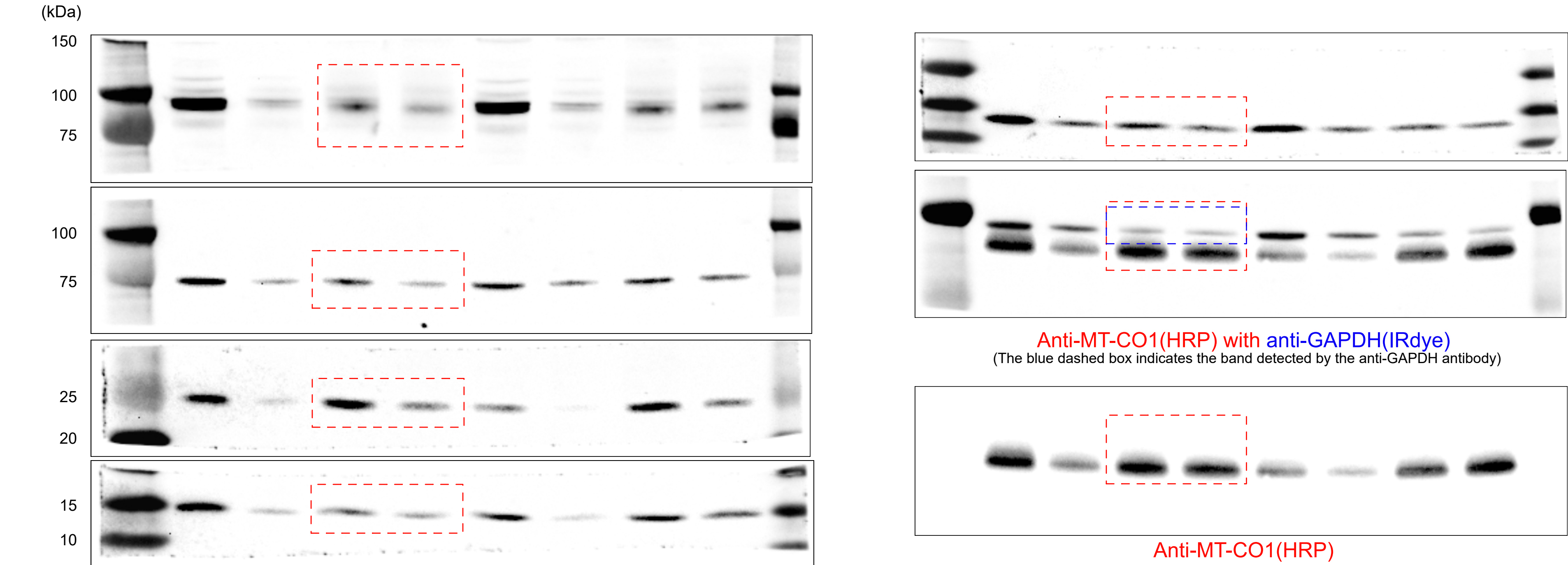

Supplement: Supplementary file 1 [file antioxidants-15-00608-s001.zip › File S1.pdf]
